# How Sports Health Professionals Perceive and Prescribe Nutritional Supplements to Olympic and Non-Olympic Athletes

**DOI:** 10.3390/ijerph191912477

**Published:** 2022-09-30

**Authors:** Floris C. Wardenaar, Daan Hoogervorst

**Affiliations:** 1 College of Health Solutions, Arizona State University, 425 N. 5th Street, Phoenix, AZ 85004, USA; 2 Independent Researcher, 2215 ZC Voorhout, The Netherlands

**Keywords:** nutritional supplements, dietary supplements, anti-doping, contamination, sports nutrition, third-party testing

## Abstract

Background: A wide range of sports health professionals provide nutritional supplement advice. We aimed to better understand the expertise, knowledge, and attitudes of sports health professionals toward nutritional supplements and third-party supplement testing. Methods: A web-based questionnaire was used to ask questions about nutritional supplement knowledge and attitudes toward the Dutch third-party supplement testing system (NZVT), about supplement efficacy, and if professionals advise these supplements, as well as which profession can be deemed the expert on nutritional supplements. Results: A total of n = 320 sports health professionals delivered input, of which 45% worked with Olympic athletes and 18% were sports dietitians. Sports dietitians were ranked as the most knowledgeable professionals about sports nutrition (80%) and nutritional supplements (74%), and a literature-based comparison showed the most favorable knowledge, attitudes, and ergogenic classifications of supplement scores for sports dietitians compared with other sports health professions. Sports health professionals working with Olympic athletes scored similar but slightly lower trends on self-reported knowledge, attitude and third-party supplement testing than sports dietitians but substantially better than professionals not working with Olympic athletes. Conclusion: Sports dietitians are seen as the absolute expert on supplements by other sports health professionals, with professionals working with Olympic athletes reporting similar trends, and other sports health professionals do have lower self-reported knowledge and preferred attitudes toward effective and safe use of dietary supplements.

## 1. Introduction

The use of nutritional supplements (NSs) is high among elite and non-elite athletes (≥97% at any point during their career and ≥85% during the last 4 weeks before filling out the questionnaire) [1], and although they often defer to family [2,3,4] and friends [3,4,5] for advice, the sports dietitian is often seen as the preferred source [6,7]. For example, athletes with access to a sports dietitian report better informed nutritional supplement choices [1] and/or better dietary habits in general [8]. On the other hand, most athletes may only have limited or no access to a designated sports dietitian [9,10]. In reality, athletes may receive nutritional supplement recommendations from a wide range of other sports health professionals, such as physicians, coaches [2,4], athletic trainers [3,9] and many more.

Athletes have consistently reported low knowledge about (sports) nutrition and nutritional supplements, ranging from 25% to 36% for supplements and ≤52% for nutrition knowledge [11,12,13,14]; as a result, appropriate guidance in the selection and use of nutritional supplements is of the utmost importance [1]. Therefore, sports health professionals should have sufficient knowledge to provide correct advice to their athletes, but relatively little is known about the knowledge and attitudes of sports professionals toward advising nutritional supplements. The knowledge of sports health professionals is ranging from 36% of coaches scoring adequate for knowledge [13], up to 81% correct answers for a nutrition knowledge test in a different group of coaches [15]. In addition, athletic trainers (71%) and strength and conditioning coaches (83%) have been reporting adequate nutrition knowledge [13]. However, sports coaches (55–69%) have also been reporting lower nutritional knowledge [16].

Multiple factors may influence why sports health professionals are more likely to recommend nutritional supplements: (1) Clients may expect from certain professions, such as athletic trainers [9] or personal trainers, that they will be able to provide nutrition care within their recommended scope of practice [17,18]. (2) Increased professional experience has been associated with higher confidence levels in personal nutrition knowledge and therefore more favorable attitudes toward providing nutrition advice [19]. (3) Self-perceived knowledge may not match actual knowledge; this results in an overestimation of one’s own ability to give good advice and may, for example, be influenced by personal experience, as the use of specific supplements influences the willingness to recommend supplements [20].

When many sports professionals are actively involved in advising or signaling nutritional supplement use of athletes, this results in a shared responsibility for athletes’ health and safety. Therefore, it should be encouraged that sports health professions have insight into each other’s standard operating procedures [7], as well as their knowledge and attitudes toward nutritional supplements. Although athletes may perceive the nutrition knowledge of athletic trainers as strong [9], beliefs on effectivity—for example, on alternative supplements, such as herbal supplements—may differ among professions [21]. This may influence the nutritional supplement advice that athletes receive. In addition, even dietitians, who have been consistently positioned as the nutrition experts [6], may report a lack of supplement familiarity [22]. As such, science-based education and discussion on the efficacy of nutritional supplements needs to take place across the whole range of sports health professions.

To ensure safe nutritional supplement use, sports health professionals need to be aware of the risks of using supplements, as well as the options for using safe third-party tested supplements. Contaminants have been found in 18.8% of nutritional supplements sold in the US [23], and recently, 38% of tested supplements contained undeclared doping substances in the Netherlands [24]. As a result, in the U.S., the number of doping violations with sanctions associated with supplements ranges up to 17% of total cases per year and has averaged around 9% of all doping cases annually since 2006 [25]. Despite instituting strict liability for drug testing in many sports organizations and providing options for safe supplement use, such as purchasing third-party tested supplements, only 50–80% of athletes report using third-party tested supplements [26,27]. As sports health professionals are important influencers in the athlete decision-making process, it is important to better understand their knowledge and attitudes toward health and/or doping risks of nutritional supplement use.

Therefore, we aimed to better understand how sports health professionals perceive and prescribe nutritional supplements while working with Olympic or non-Olympic athletes. For this reason, we assessed the self-reported knowledge and attitudes of Dutch sports health professionals toward the use of a commonly used third-party supplement testing system in the Netherlands. This descriptive study highlights differences for a wide range of sports health professionals, working with Olympic/Paralympic athletes, as well as differences between these professionals (i.e., sports dietitians vs. other professions) based on their years of experience working in sports.

## 2. Materials and Methods

### 2.1. Study Design and Population

Data collection for this descriptive study took place between 2013 and 2015, using a web-based questionnaire (The Qualtrics Research Suite, 2013, Provo, UT, USA) with a total duration of 30–45 min. Responses from sports health professionals (i.e., professionals working with athletes as listed in Table 1), were obtained during multiple events (professional conferences and workshops) in the Netherlands, but the actual size of the population receiving the invitation was not registered. All professionals (≥18 years old and self-identifying as sports health professionals) delivering a complete set of answers to the questions were included in the data analysis. The study was conducted according to the declaration of Helsinki [28], and before proceeding to the actual questionnaire, participants were made aware that the aggregated results would be published, but no written informed consent was obtained from participants. Data analysis was approved by the Institutional Review Board at Arizona State University (STUDY00016538).

### 2.2. Questionnaire

The questionnaire, previously described by Wardenaar et al. (2017) [1], was used as a basis for sports professionals covering demographics, as well as sixteen questions about knowledge and attitudes toward the Dutch third-party NZVT system (Nederlands Zekerheidssysteem Voedingssupplementen in de Topsport). Knowledge questions 1 and 2 were formatted to a 5-point Likert scale (range: 1–5), and questions 3–8 to yes/no/not sure responses, with 5–8 asking if a factual statement is correct. Attitude questions 1–6 were formatted to a similar Likert scale, and questions 7 and 8 to yes/no/not sure. For readability, the scale for the attitude Q4 item was reversed (the traditional statement was: “The topic of contaminated NS is irrelevant to me, and therefore, it is not of interest to me”). The questions described above are displayed in Table 2 and Table 3, respectively. Likert scales for agree and strongly agree were combined and displayed as percentages. Additionally, questions were altered to ask about sports professionals’ perceptions about the efficacy of nutritional supplements and if they advised specific supplements (the full list of pre-defined supplements, categorized as dietary supplements, sports foods and ergogenic supplements [1], are listed as part of Appendix A Table A1). Finally, sports professionals were asked about their self-perceived expertise and that of other professions in the area of nutritional supplements.

### 2.3. Data Analysis

The data were analyzed using SPSS (IBM SPSS Statistics, version 28, Armonk, NY, USA). Sports health professional characteristics (i.e., sex, education, profession, working with Olympic or Paralympic athletes [from now on indicated as Olympic athletes] or not) and years of experience are reported as a percentage (%) and frequency (n). Knowledge and attitude outcomes were reorganized as dichotomous outcomes, in which the not agree-not disagree score, or the unsure option, was added to the negatively perceived outcome. Question four of the knowledge section in Table 2 addressed familiarity with the NZVT system; the analysis of knowledge questions 5–8 and attitude questions 7–8 were based on the responses of the athletes familiar with the NZVT (Y on question 4). Chi-square analysis was applied to determine differences between categorical variables for sports professionals working with Olympic athletes and non-Olympic athletes and with years of experience (≤5 years vs. 6 years or more) and demographic outcomes. Categorical differences were applied to original uncategorized answers using Mann–Whitney U Tests. The significance was set for *p*-value ≤ 0.05.

## 3. Results

### 3.1. Characteristics

Out of n = 387 we included a total of n = 320 sports health professionals that answered all questions, 45% (n = 144) worked with Olympic athletes, and sports dietitians represented 18% (n = 59) of the total population. The education level of professionals working with Olympic athletes was significantly higher than professionals working with non-Olympic athletes (*p* < 0.001), but no significant difference was seen for the education level of the sports dietitians versus the other professions (*p* = 0.082). The profession-based sample distribution differed for professionals working with Olympic athletes and when comparing sports dietitians with other professions (*p* < 0.001), but not for years of experience (*p* = 0.522).

### 3.2. Self-Reported Knowledge and Attitudes

Table 2 describes the outcomes of the self-reported knowledge toward NS and third-party testing (i.e., the NZVT-system). The trends for the answers of professionals working with Olympic athletes (including 66% of the sports dietitians) are very similar to the answers of the sports dietitians alone.

Less than 35% of the professionals know about the effect of contamination on a supplement. Professionals working with Olympic status athletes reported a higher self-reported knowledge for this proposition than professionals working with lower level athletes (41% vs. 39%, *p* = 0.033); when stratifying data for sports dietitians and other professionals, this was 36% vs. 25% (*p* = 0.005). A total of 68% of all professionals believed that contaminated NS could lead to a positive doping test; however, professionals working with Olympic status athletes (80%) were more convinced of this than professionals working with lower level athletes (57%, *p* < 0.001). A total of 88% of the sports dietitians vs. 63% of the other professionals’ report that contamination could lead to a positive doping test (*p* = 0.003). Only 44% of all professionals were familiar with ingredients that were listed on the World Anti-Doping Agency (WADA) doping list; again, professionals working with Olympic status athletes (55%) vs. the ones not working with Olympic athletes (35%) were more familiar with this statement (*p* < 0.001). Again, sports dietitians alone (66%) reported higher familiarity with WADA listed ingredients than the other professions (39%, *p* < 0.001). A total of 47% of all professionals reported to be familiar with the NZVT, but especially the sports health professionals working with Olympic status athletes reported to be more familiar with the NZVT system (69% vs. 30%, *p* < 0.001). Sports dietitians reported 88% familiarity with NZVT vs. 38% of the other professions (*p* < 0.001). Of the professionals familiar with the NZVT, the professionals working with Olympic athletes were more aware that NZVT testing was not suggesting efficacy of a supplement (67% vs. 47%, *p* = 0.017), with sports dietitians reporting higher than other professionals (78% vs. 51%, *p* = 0.007). The knowledge about the NZVT logo was around 45% or less, whereas knowledge about third-party testing certifications not being completely water tight in ensuring supplements to be free from doping was on average 72% (*p* > 0.05).

Table 3 describes the outcomes of the self-reported attitudes toward NS and third-party testing (i.e., the NZVT-system). Almost all professionals (86%) wanted to know the risks associated with incorrect NS use, 89% finds supplement contamination unacceptable, and 87% would like to know what the risks are of taking a contaminated NS, without differences for professionals working with or not working with Olympic athletes, or between sports dietitians and other professions (*p* > 0.05).

More than half (53%) of the participants reported that the topic of contaminated NSs was relevant for them, with higher numbers for professionals working with Olympic status athletes (73%) than professionals working with non-status athletes (38%, *p* < 0.001).

This number was 78% for the sports dietitians vs. the other professions with 48% (*p* < 0.001). Almost half of the participants (49%) reported to have a clear idea of the effect of misusing NS, with professionals working with Olympic status athletes reporting higher values than those working with non-Olympic status athletes (60% vs. 40%, *p* < 0.001), and sports dietitians vs. other professions (73% vs. 44%, *p* < 0.001). Similar numbers (50%) were reported by professionals on how contaminated supplements could result in a positive doping test (67% vs. 36%, *p* < 0.001) and sports dietitians vs. other professions (71% vs. 45%, *p* < 0.001). A total of 89% of the professionals working with status athletes used the NZVT system while purchasing NSs in comparison to 63% of the professionals that do not work with status athletes (*p* < 0.001). A total of 92% of the sports dietitians reported its use vs. 74% of the others familiar with NZVT. Of the professionals familiar with NZVT, 71% (no Olympic status difference, *p* = 0.054) reported being satisfied with the system, but sports dietitians were less satisfied (59%) than other professions (78%, *p* = 0.007).

When stratifying data for experience (less or more than 6 years of experience) the only difference was self-reported familiarity with ingredients listed by WADA. The professionals with >6 years of experience reported they were more familiar with the WADA list than their colleagues with less experience (51% vs. 33%, *p* = 0.002). Further, the less experienced professionals reported to be more satisfied (86% vs. 63%) with the NZVT system in comparison to the group with more experience (*p* = 0.004). No other differences were seen for experience in relation to the questions reported in Table 2 and Table 3.

### 3.3. Professions and Sports Nutrition Knowledgeability

The largest number of sports health professionals determined the sports nutritionist or sports dietitian as the most knowledgeable in the field of general sports nutrition advice (80%), followed by the coach (6%) and sport physician (5%), who were assigned as the preferred professional. In addition (Table 4), a similar percentage of professionals marked the sports nutritionist or sports dietitian as most knowledgeable in the field of sports nutrition supplements and nutritional preparations (74%), followed by the sports physician (10%) and the coach (9%).

Despite the overwhelming support for the sports dietitian, there was almost for each profession a substantial group of professionals that identify themselves as the expert in the field of general sports nutrition and/or nutritional supplements, with sport scientists reporting this most frequently (38% and 36%), followed by coaches (26% and 31%) and caregivers (19% and 29%).

The self-reported knowledgeability (Table 5) was normally the highest for general nutrition, followed by sports nutrition and nutritional supplements, with some small exceptions. Overall, sports dietitians, sports scientists, and caregivers were the most positive about their personal knowledgeability.

### 3.4. Classification of Ergogenic Effect of Supplements and Supplement Advise

The left side of Table 6 reports if sports health professionals as a group and stratified for sports dietitians and other professions think if the pre-determined nutritional supplements have a performance enhancing (ergogenic) effect while classified as dietary supplement, sports food, or ergogenic supplement.

One-third of the professionals reports multivitamins (31%) as ergogenic, followed by fish oil (29%), and magnesium (27%), but a substantially lower number of sports dietitians (7%) vs. other professions (31%) defines magnesium as an ergogenic substance (*p* < 0.001). In almost all cases, the reported sports foods are more frequently determined as ergogenic by sports dietitians than other professions (recovery drinks: 86% vs. 56%, CHO-electrolyte.

Additionally, we asked sports health professionals if they advised these specific supplements to their athletes. As shown by the right side of Table 6, in almost all cases sports dietitians advised supplements significantly more frequently to their athletes than other sports professions (with differences ranging between 11% and 64%, with *p*-value ranging from <0.05 to <0.001), except for sodium bicarbonate (14% vs. 9%, *p* = 0.144), and supplements that are not directly listed as ergogenic, such as magnesium (31% vs. 38%, *p* = 0.147), anti-oxidants (24% vs. 17%, *p* = 0.090), zinc (17% vs. 15%, *p* = 0.364), amino acids (30% vs. 30%, *p* = 0.481), and MCTs (7% vs. 14%, *p* = 0.069). Sports dietitians most often advised CHO-electrolyte beverages (95%), recovery drinks (91%), and multivitamins (91%), whereas other professionals most often advised recovery drinks (59%), multivitamins (49%), and CHO-electrolyte beverages (43%), *p* < 0.001. Finally, to ensure focus on the most important results, not all reported supplements have been included in Table 6.

## 4. Discussion

This study aimed to better understand how sports health professionals perceive and prescribe nutritional supplements while working with Olympic or non-Olympic athletes. Sports dietitians were ranked by their colleague sports health professionals as the most knowledgeable profession about sports nutrition and nutritional supplements. Sports health professionals working with Olympic athletes reported similar outcomes but slightly less favorable outcomes as sports dietitians, but better than their counterparts working with non-Olympic athletes.

The mindset of professionals working with Olympic status athletes with regard to the theme of nutritional supplements was comparable to the sports dietitian, but sports dietitians provided a little more often the desired answers in relation to nutritional supplement third-party testing (i.e., NZVT). Sports dietitians were ranked as the most skilled by their peers, but across the board there was a small group of sports health professionals, other than the sports dietitians, who rated themselves as being the most skilled expert. Sports dietitians confirmed their expertise indirectly by reporting a higher degree of knowledge, for example by more often correctly classifying ergogenic supplements compared with other professions. When it comes to advice, the sports dietitian advises nutritional supplements more frequently in most cases, but not always, and in that case, it always concerns nutritional supplements that lack scientific background for its ergogenic effect. Although professionals working with Olympic athletes show similar trends in their reporting, sports dietitians report the highest knowledge levels and most desirable attitudes. As other professions may have opposite ideas about nutritional supplements that should be recommended, this could lead to interprofessional disconnect, resulting in different communication flows between sports health professionals, or to incorrect or sub-optimal nutritional supplement recommendations in a team where a sports dietitian is missing.

Recently, it has been suggested that there are specific situations where performance may be compromised by a strict food only approach [29]. Beneficial supplements include CHO, protein, iron, caffeine, creatine, beta-alanine, nitrate, sodium bicarbonate, and substances related to the immune system, such as vitamin D, vitamin C, probiotics, and zinc [29], and except for sodium bicarbonate and zinc, sports dietitians more often recommend these substances than other professions. Overall, we need to appreciate that even though these data were collected between 2012 and 2015, there was already strong scientific evidence for use of these supplements in specific situations in sport when using evidence-based protocols [30]. At that moment in time, a systematic review was published that supported supplementing sodium bicarbonate supplementation to increase power capacity during high intensity races [31], but the number of sports for which supplementation may contribute to substantial performance enhancement is relatively small. In combination with the substantial side effects associated with sodium bicarbonate only a limited number of professionals advising this supplement was to be expected. Although multiple authors wrote about the effect of zinc to minimize exercise-induced immunosuppression in athletes, it is unlikely to be of benefit to athletes unless they are zinc deficient [32], which can be an explanation for the relatively low number of sports health professionals advising zinc.

It is important to mention that there was not a 100% match between the reported ergogenic capacity of the nutritional supplements scored by professionals and expert literature consensus. The difference was smaller for sports dietitians than other professions, suggesting that sports dietitians indeed, as suggested in this study by their colleagues, are more knowledgeable on the topic of sports nutrition supplements. Knowledge transfer may not always be as effective; it has been suggested that the most effective knowledge transfer tool is developing a community of practice, followed by mentoring, storytelling, succession plans, and lastly coaching [33]. This means that teams should consider how sports dietitians and other sports health professionals improve their knowledge on this topic. The basis for the content of this discussion should be the available international consensus documents, and the information of national sports sciences bodies and national dietetic organizations. In this case, specifically when focusing on safe nutritional supplement, we suggest the use of practical tools such as the flow chart described by Close et al. (2022) [29]. The tool, will help to guide discussion between sports health professionals about the need of specific supplements. Taking in account that there are many challenges, like problems with time management [34] and/or limited professional support [35], sports health professionals need to reserve time as part of their team work to agree on the topic of meaningful and safe supplement usage to ensure athletes are served with the highest quality of advice [36].

As far as we know our study is the first to ask a wide range of sports health professionals about their knowledge and attitudes toward athlete nutritional supplement use (including the supplement efficacy and the need to advise these supplements) and the use of third-party tested nutritional supplement use. It clearly shows the difference in nutritional supplement expertise among professions, in which sports dietitians can be considered the expert, as previously suggested [6,7], but professionals working with Olympic athletes are more frequently reporting well-informed outcomes than those working with non-Olympic athletes. A recent data collection revealed that the preferred source of US student-athletes to ask about nutritional supplements differed between Division I and Division III, in favor of a sports dietitian (58% for DI) vs. athletic trainer (52% for DIII [37]. Hamilton et al. (2022) suggest that this was due to the fact that a sports dietitian was not employed at the DIII level.

Another study reporting differences between professional and amateur rugby players reported that their preferred source of nutritional supplement information was their sport trainer (17%), followed by friends (14%) and then a sports dietitian (12%) [38], while it was also reported that family and friends (20%), then strength trainer (14%) and teammates (11%), were the preferred source [39]. This illustrates that athletes may receive advice from a wide range of more or less qualified individuals, and that there may be different scenarios in which athletes may receive supplement advice [36]. This includes the scenario in which the sports dietitian is the primary source of information, but when a sports dietitian is not available, another professional provides this advice; also, there is the situation where athletes may receive advice from multiple professionals. The last option can especially lead to interprofessional disconnect, as part of knowledge difference or as reflected by our data, because some professionals feel they are more of an expert than the sports dietitian [36]. Overall, we want to stress the importance of increasing supplement knowledge in all sports health professionals that provide advice, and we recommend that the sports health professionals that work together reserve time to agree on the need or importance of specific nutritional supplements and the desired periodization, timing, frequency, and dosage of these supplements.

When comparing the results of the current study for using the preferred third-party testing system in the Netherlands (i.e., NZVT) with the self-reported use of athletes. The professionals score substantially higher than the athletes, reporting an 80% overall compliance up to 89% for professionals working with Olympic athletes and 92% for sports dietitians vs. 80% of the athletes with an Olympic status and 50% of the athletes without Olympic status [26]. Although there were no data to confirm improved compliance, we assume that an increase in professional compliance should also result in improved future athlete compliance.

Around 65% of athletes have reported use of multivitamin and mineral supplements [1,27], which is slightly higher than advised by the total group of sports health professionals (57%), but lower than advised by sports dietitians (91%). A total of 46% of Dutch athletes reported the use of sports drinks and recovery drinks [1], while 65% and 53%, respectively of sports health professionals report advising these products. Finally, ergogenic supplements were reported to be used by Dutch athletes: 23% (caffeine), 19% (creatine), and 16% (nitrate) [1], while these substances were advised by sports health professionals in the current study for 30%, 32%, and 25%, respectively. In contrast to the use of multivitamin and mineral supplements in which the self-reported use by athletes was higher, the use of the other supplements was around 10% lower than the reported percentage of sports health professionals reporting to advise these supplements.

This study is not without limitations, as the data was collected between 2013 and 2015, and therefore the situation does not necessarily reflect the current situation. Further, the data consist only of self-reported knowledge and attitudes, focusing on sports nutrition supplements, and no validated knowledge scale was used; therefore, the results may not be generalizable for other elements of the athlete diet. Further, the study recruited a convenience sample of different professionals, as a result some professions such as strength and conditioning coaches were underrepresented. At the same time the sports health professionals may or may not have worked together; the current dataset does not allow further assessment of interactions between professions within a specific sport, team, or the impact that their (lack of) collaboration has on the actual athlete behavior. We suggest that future research looks more into this side of sports nutrition collaboration. The data collection may be subject to reporting bias as the professionals filling out this questionnaire most of the time expressed interest in dietary supplements (as the questionnaire was sent out before participating in workshop sessions during multiple professional conferences). Finally, we need to consider that six out of eight knowledge questions were yes or no questions, which gives the professional a 50% chance of guessing the good answer without really knowing the right answer.

## 5. Conclusions

In conclusion, sports dietitians are seen as the absolute expert by other sports health professionals, but their self-reported knowledge and attitude is not always in full compliance with the sports nutrition expert consensus. Professionals working with Olympic athletes report similar trends for safe use of dietary supplements as sports dietitians alone. Although sports dietitians report a much higher frequency for advising specific nutritional supplements than others professions, there is a substantial group of other professionals that advise these products as well. While other professions seem to have less expertise on the ergogenic effect of nutritional supplements, sports dietitians and other sports health professionals should be aware of the potential disconnect in how athletes are advised about using nutritional supplements.

This results in the following practical applications: (1) Knowledge of sports health professionals about third-party testing is low, therefore they should increase their knowledge level about safe nutritional supplement use to ensure that the right information is conveyed to athletes. (2) Increasing collaboration between disciplines, both inter- and intradisciplinary, should be improved to facilitate better athlete guidance towards to safe nutritional supplement use.

## Figures and Tables

**Table 1:** Respondent characteristics (Percentage or N or percentage), reported as group total and stratified for professionals working or not working with Olympic athletes and for profession.

	Group Total	Working with Olympic Athletes			Profession		
		Yes	No	*p*-Value	Sports Dietitian	Other Profession	*p*-Value
N	320	144	176		59	261	
**Sex M/F**	174/146	75/69	99/77	0.457	2/57	172/89	**<0.001**
Highest achieved education level *				**<0.001**			0.082
Trade school	20 (63)	16 (23)	23 (40)		2 (1)	24 (62)	
Bachelor (BS)	44 (140)	42 (60)	46 (80)		76 (45)	36 (95)	
Master/Doctorate (MS/PhD)	30 (97)	41 (59)	23 (38)		20 (12)	33 (85)	
**Profession**				**<0.001**			**<0.001**
Different	64	10	54		0	64	
Sports dietitian	59	39	20		59	0	
Coach	43	18	25		0	43	
Sports physician	39	27	12		0	39	
Masseur	33	13	20		0	33	
Physiotherapist	26	15	11		0	26	
Sport scientist	17	4	13		0	17	
Caregiver	17	8	9		0	17	
Sport manager	12	5	7		0	12	
Mental coach	8	5	3		0	8	
Strength coach	2	0	2		0	2	

The highest achieved education has been reported in three different categories: Trade school (translated from Dutch: MBO), Bachelor (BS) (in Dutch: HBO), and Master/Doctorate (MS/PhD) (in Dutch: WO). Significant differences are expressed in bold font. * Numbers add not completely up to 100% as high school (in Dutch: vmbo, havo or vwo) was not included in this table.

**Table 2:** Self-reported knowledge about contaminated nutritional supplements (NSs) and dietary supplement third-party testing (% and N) for the total group, professionals working or not working with Olympic athletes, and sports dietitians vs. other professions.

			Working with Olympic Athletes			Profession		
Questions		Total	Yes	No	*p*-Value	Sports Dietitian	Other Profession	*p*-Value
No. (type of scale)		% (N)	% (N)	% (N)		% (N)	% (N)	
**Knowledge**								
1 (L)	Contaminated NSs have no small effect or positive effect at all.	35 (111)	41 (59)	39 (52)	**0.033**	36 (26)	25 (85)	**0.005**
2 (L)	Use of contaminated NSs can lead to a positive doping test.	68 (216)	81 (116)	57 (100)	**<0.001**	88 (52)	63 (164)	**0.003**
3 (Y/N)	Are you familiar with NSs of which ingredients are listed on the WADA doping list?	44 (141)	55 (79)	35 (62)	**<0.001**	66 (39)	39 (102)	**<0.001**
4 (Y/N)	Are you familiar with the NZVT system?	47 (151)	69 (99)	30 (52)	**<0.001**	88 (52)	38 (99)	**<0.001**
5 (Y/N)	Products listed on the NZVT list are not tested on efficacy. *	61 (89)	67 (66)	47 (23)	**0.017**	78 (40)	51 (49)	**0.007**
6 (Y/N)	Only products with the NZVT logo on the label are always tested. *	53 (78)	44 (43)	47 (23)	0.295	61 (31)	49 (47)	0.106
7 (Y/N)	The absence of the NZVT logo on the label indicates that only certain batches of the NSs are tested. *	43 (63)	46 (45)	37 (18)	0.290	59 (30)	34 (33)	0.155
8 (Y/N)	The NZVT system does provide a 99% guarantee that tested NSs are free from doping substances. *	72 (106)	74 (72)	69 (34)	0.604	88 (45)	64 (61)	0.111

Significant differences are indicated in bold. Questions 1–4 are based on the total population (n = 601, with n = 80–85 missing answers). Questions 5–8 were answered by 259 of those that answered Y (n = 370) on question 4, which asked if they were familiar with the NZVT system. L = Likert scale; Y/N = Yes/No. Asterisks indicate that results are based on questions that were asked only when participants answered to be familiar with the NZVT system (N = 147). Significant differences are expressed in bold font.

**Table 3:** Self-reported attitude about contaminated nutritional supplements (NSs) and dietary supplement third-party testing (% and N) for the total group, professionals working or not working with Olympic athletes, and sports dietitians vs. other professions.

			Working with Olympic Athletes			Profession		
Questions		Total	Yes	No	*p*-Value	Sports Dietitian	Other Profession	*p*-Value
No. (type of scale)		% (N)	% (N)	% (N)		% (N)	% (N)	
**Attitude**								
1 (L)	I would like to know what the risk of incorrect use of NSs is.	86 (274)	89 (128)	83 (146)	0.133	83 (49)	86 (225)	0.127
2 (L)	I find the idea of using a contaminated NS without knowing this unacceptable.	89 (285)	90 (129)	89 (156)	0.787	90 (53)	89 (232)	0.478
3 (L)	I would like to know what the risk is of taking a contaminated NS.	87 (278)	89 (128)	85 (150)	0.335	83 (49)	88 (229)	0.101
4 (L)	The topic of contaminated NSs is relevant to me, and therefore, it is of interest to me.	53 (171)	73 (105)	38 (66)	**<0.001**	78 (46)	48 (125)	**<0.001**
5 (L)	I have a clear idea what the effect is of misusing NSs.	49 (157)	60 (87)	40 (70)	**<0.001**	73 (43)	44 (114)	**<0.001**
6 (L)	I have a clear idea how a contaminated NS can result in a positive doping test.	50 (159)	67 (96)	36 (63)	**<0.001**	71 (42)	45 (117)	**<0.001**
7 (Y/N)	Do you use the NZVT list while purchasing products? *	80 (118)	89 (87)	63 (31)	**<0.001**	92 (47)	74 (71)	**0.004**
8 (Y/N)	Are you satisfied with the NZVT system? *	71 (105)	66 (65)	82 (40)	0.054	59 (30)	78 (75)	**0.007**

Asterisks indicate that results are based on questions that were asked only when participants answered that they were familiar with the NZVT system (N = 147), significant differences are expressed in bold font.

**Table 4:** Participating sports health professionals identified the profession with the ultimate expertise on sports nutrition and supplements.

	Dietitian	Coach	Sports Physician	Masseur	Physio-Therapist	Sports Scientist	Caregiver	Sports Manager	Mental Coach	Strength Coach
General sports nutrition (n = 249)	80% (n = 199)	6% (n = 15)	5% (n = 12)	2% (n = 5)	2% (n = 6)	2% (n = 6)	1% (n = 3)	1% (n = 2)	0% (n = 0)	1% (n = 1)
Nutritional supplements (n = 223)	74% (n = 165)	9% (n = 20)	10% (n = 23)	1% (n = 1)	2% (n = 5)	2% (n = 4)	2% (n = 4)	1% (n = 1)	0% (n = 0)	0% (n = 0)

The percentage for each profession was based on each sports health professional’s first choice.

**Table 5:** Self-reported knowledgeability for general nutrition, sports nutrition, and nutritional supplements for each sports health profession.

	Dietitian (n = 59)	Coach (n = 43)	Sports Physician (n = 39)	Masseur (n = 33)	Physio-Therapist (n = 26)	Sports Scientist (n = 17)	Caregiver (n = 17)	Sports Manager (n = 12)	Mental Coach (n = 8)	Strength Coach (n = 2)	Other (n = 64)
General nutrition	94%	72%	67%	50%	50%	83%	68%	58%	64%	100%	64%
Sports nutrition	82%	53%	56%	45%	43%	50%	69%	50%	55%	100%	35%
Nutritional supplements	62%	42%	46%	40%	23%	50%	42%	25%	27%	75%	47%

Self-reported knowledgeability was reported on a Likert-scale from 1–5 (from totally disagree to totally agree), and then calculated as percentage in which each element on the scale represented 20%.

**Table 6:** Ergogenic effect and advising of nutritional supplements by sports health professionals.

	Do You Think This Supplement Has an Ergogenic Effect?				Do You Advise This Supplement to Your Athletes?			
	Total (n = 305)	Dietitian (n = 58)	Other (n = 247)	*p*-Value	Total (n = 305)	Dietitian (n = 58)	Other (n = 247)	*p*-Value
**Dietary supplements (i.e., vitamins, minerals, and essential fatty acids)**								
Multivitamin	31 (94)	33 (19)	30 (75)	0.363	57 (174)	91 (53)	49 (121)	**<0.001**
Fish oil	29 (87)	33 (19)	28 (68)	0.207	45 (136)	79 (45)	37 (91)	**<0.001**
Magnesium	27 (81)	7 (4)	31 (77)	**<0.001**	37 (113)	31 (18)	38 (95)	0.147
Vitamin D	22 (66)	36 (21)	18 (45)	**0.001**	33 (99)	71 (41)	23 (58)	**<0.001**
Iron	19 (59)	16 (9)	20 (50)	0.207	30 (90)	50 (29)	25 (61)	**<0.001**
Vitamin C	16 (48)	14 (8)	16 (40)	0.326	25 (76)	36 (21)	22 (55)	**0.014**
Vitamin B-complex	16 (48)	16 (9)	16 (39)	0.480	24 (72)	40 (23)	20 (49)	**<0.001**
Anti-oxidants	13 (40)	12 (7)	13 (33)	0.397	18 (55)	24 (14)	17 (41)	0.090
Vitamin B12	14 (43)	8 (5)	15 (38)	0.092	18 (55)	28 (16)	16 (39)	**0.018**
Zinc	12 (35)	7 (4)	13 (31)	0.113	16 (48)	17 (10)	15 (38)	0.364
**Sports foods (i.e., sports drinks, protein shakes, energy bars and isolated carbohydrate or protein supplements)**								
Recovery drink	61 (185)	86 (49)	56 (136)	**<0.001**	65 (195)	91 (52)	59 (143)	**<0.001**
CHO-electrolyte beverage	46 (139)	84 (48)	38 (91)	**<0.001**	53 (159)	95 (54)	43 (105)	**<0.001**
Protein drink	44 (130)	46 (26)	43 (104)	0.341	44 (101)	54 (31)	41 (101)	**0.038**
Energy bar	41 (124)	67 (38)	36 (86)	**<0.001**	48 (144)	79 (45)	41 (99)	**<0.001**
CHO-PRO electrolyte beverage	36 (108)	67 (38)	29 (70)	**<0.001**	37 (112)	74 (42)	29 (70)	**<0.001**
Energy gel	35 (104)	72 (41)	26 (63)	**<0.001**	35 (105)	74 (42)	26 (63)	**<0.001**
Protein bar	29 (86)	32 (18)	28 (68)	0.289	30 (89)	39 (22)	28 (67)	**0.049**
**Ergogenic supplements (i.e., supplements other than above, claiming to aid in performance, recovery or health)**								
Creatine	39 (115)	67 (38)	32 (77)	**<0.001**	32 (95)	63 (36)	25 (59)	**<0.001**
Caffeine	36 (107)	56 (32)	32 (75)	**<0.001**	30 (88)	53 (30)	24 (58)	**<0.001**
Amino acids	31 (91)	32 (18)	30 (73)	0.403	30 (99)	30 (17)	30 (72)	0.481
Probiotics	13 (37)	40 (23)	5 (13)	**<0.001**	26 (75)	77 (44)	13 (31)	**<0.001**
Beetroot/dietary nitrate	29 (85)	54 (31)	23 (54)	**<0.001**	25 (74)	49 (28)	19 (46)	**<0.001**
B-alanine	18 (54)	44 (25)	12 (29)	**<0.001**	13 (38)	32 (18)	8 (20)	**<0.001**
Sodium bicarbonate	15 (44)	32 (18)	11 (26)	**<0.001**	10 (30)	14 (8)	9 (22)	0.144
MCT	11 (32)	4 (2)	13 (31)	**0.023**	13 (39)	7 (4)	14 (35)	0.069

Beverages: 84% vs. 38%, energy bars: 67% vs. 36%, CHO-PRO electrolyte beverages: 67% vs. 29%, and energy gels: 72% vs. 26%, *p* < 0.001). Protein drinks were reported to be ergogenic by 44% (*p* = 0.341), and a protein bar by 29% (*p* = 0.289). Except for amino acids (32% vs. 30%, *p* = 0.403) and medium-cyclic-triglycerides (MCT, 4% vs. 13%, *p* < 0.001), sports dietitians more often than other professions determine the listed ergogenic supplements as an ergogenic (creatine: 67% vs. 32%, caffeine: 56% vs. 32%, probiotics: 40% vs. 5%, beetroot/dietary nitrate: 54% vs. 32%, B-alanine: 44% vs. 12%, sodium bicarbonate: 32% vs. 11%, *p* < 0.001). Significant differences are expressed in bold font.

## Data Availability

Not applicable.

## Appendix A. Contains the Full Pre-Defined List of Nutritional Supplements Questioned after the Reference Section

**Table A1:** Perception of ergogenic effect of nutritional supplements and if professionals advise these nutritional supplements for the total groups of sports health professionals and stratified for sports RDs and other professionals.

	Do You Think This Supplement Has an Ergogenic Effect?				Do You Advise This Supplement to Your Athletes?			
	Total (n = 305)	Sports Dietitian (n = 58)	Other (n = 247)	*p*-Value	Total (n = 305)	Sports Dietitian (n = 58)	Other (n = 247)	*p*-Value
**Dietary supplements (i.e., vitamins, minerals, and essential fatty acids)**								
Multi vitamin	31 (94)	33 (19)	30 (75)	0.363	57 (174)	91 (53)	49 (121)	**<0.001**
Fish oil	29 (87)	33 (19)	28 (68)	0.207	45 (136)	79 (45)	37 (91)	**<0.001**
Magnesium	27 (81)	7 (4)	31 (77)	**<0.001**	37 (113)	31 (18)	38 (95)	0.147
Vitamin D	22 (66)	36 (21)	18 (45)	**0.001**	33 (99)	71 (41)	23 (58)	**<0.001**
Iron	19 (59)	16 (9)	20 (50)	0.207	30 (90)	50 (29)	25 (61)	**<0.001**
Combination vitamin	19 (59)	22 (13)	19 (46)	0.256	28 (86)	36 (21)	23 (65)	0.066
Combination mineral	20 (60)	14 (8)	21 (52)	0.106	26 (80)	31 (18)	25 (62)	0.178
Vitamin C	16 (48)	14 (8)	16 (40)	0.326	25 (76)	36 (21)	22 (55)	**0.014**
Vitamin B-complex	16 (48)	16 (9)	16 (39)	0.480	24 (72)	40 (23)	20 (49)	**<0.001**
Anti-oxidants	13 (40)	12 (7)	13 (33)	0.397	18 (55)	24 (14)	17 (41)	0.090
Vitamin B12	14 (43)	8 (5)	15 (38)	0.092	18 (55)	28 (16)	16 (39)	**0.018**
Zinc	12 (35)	7 (4)	13 (31)	0.113	16 (48)	17 (10)	15 (38)	0.364
Calcium	9 (26)	3 (2)	10 (24)	0.062	15 (47)	21 (12)	14 (35)	0.109
Folic acid	9 (26)	3 (2)	10 (24)	0.062	12 (37)	16 (9)	11 (28)	0.191
Selenium	8 (24)	2 (1)	9 (23)	**0.027**	9 (28)	2 (1)	11 (27)	**0.014**
Vitamin B6	6 (18)	3 (2)	6 (16)	0.190	7 (21)	3 (2)	8 (19)	0.126
Chrome	6 (18)	2 (1)	7 (17)	0.067	7 (20)	3 (2)	7 (18)	0.145
Vitamin A	5 (15)	2 (1)	6 (14)	0.106	5 (15)	5 (3)	5 (12)	0.461
B-carotene	5 (15)	0 (0)	6 (15)	**0.027**	5 (14)	2 (1)	5 (13)	0.124
Vitamin B1	6 (19)	5 (3)	6 (16)	0.356	5 (16)	3 (2)	6 (14)	0.248
Vitamin B2	6 (17)	3 (2)	6 (15)	0.217	5 (14)	3 (2)	5 (12)	0.323
Vitamin E	4 (12)	0 (0)	5 (12)	**0.044**	5 (14)	3 (2)	5 (12)	0.323
Other oils	4 (13)	2 (1)	5 (12)	0.146	6 (17)	4 (2)	6 (15)	0.219
**Sports foods (i.e., sports drinks, protein shakes, energy bars and isolated carbohydrate or protein supplements)**								
Recovery drink	61 (185)	86 (49)	56 (136)	**<0.001**	65 (195)	91 (52)	59 (143)	**<0.001**
Beverage with carbs and electrolytes	46 (139)	84 (48)	38 (91)	**<0.001**	53 (159)	95 (54)	43 (105)	**<0.001**
Energy bar	41 (124)	67 (38)	36 (86)	**<0.001**	48 (144)	79 (45)	41 (99)	**<0.001**
Protein drink	44 (130)	46 (26)	43 (104)	0.341	44 (101)	54 (31)	41 (101)	**0.038**
Beverage with carbs, electrolytes and protein	36 (108)	67 (38)	29 (70)	**<0.001**	37 (112)	74 (42)	29 (70)	**<0.001**
Energy gel	35 (104)	72 (41)	26 (63)	**<0.001**	35 (105)	74 (42)	26 (63)	**<0.001**
Lemonade syrup	27 (82)	56 (32)	21 (50)	**<0.001**	31 (93)	61 (35)	24 (58)	**<0.001**
Protein bar	29 (86)	32 (18)	28 (68)	0.289	30 (89)	39 (22)	28 (67)	**0.049**
Grape sugar	26 (79)	30 (17)	26 (62)	0.259	30 (90)	33 (19)	29 (71)	0.278
Energy drink	28 (83)	49 (28)	23 (55)	**<0.001**	27 (82)	53 (30)	22 (52)	**<0.001**
Maltodextrin * N = 84	55 (46)	78 (21)	44 (25)	**0.002**	14 (41)	35 (20)	9 (21)	**<0.001**
Ribose	5 (14)	0 (0)	6 (14)	**0.032**	4 (13)	4 (2)	5 (11)	0.365
**Ergogenic supplements (i.e., supplements other than above, claiming to aid in performance, recovery or health)**								
Creatine	39 (115)	67 (38)	32 (77)	**<0.001**	32 (95)	63 (36)	25 (59)	**<0.001**
Caffeine	36 (107)	56 (32)	32 (75)	**<0.001**	30 (88)	53 (30)	24 (58)	**<0.001**
Amino acids	31 (91)	32 (18)	30 (73)	0.403	30 (99)	30 (17)	30 (72)	0.481
Probiotics	13 (37)	40 (23)	5 (13)	**<0.001**	26 (75)	77 (44)	13 (31)	**<0.001**
Beetroot/dietary nitrate	29 (85)	54 (31)	23 (54)	**<0.001**	25 (74)	49 (28)	19 (46)	**<0.001**
Glucosamine-chondrotoïne	10 (28)	9 (5)	10 (23)	0.415	15 (44)	16 (9)	15 (35)	0.423
B-alanine	18 (54)	44 (25)	12 (29)	**<0.001**	13 (38)	32 (18)	8 (20)	**<0.001**
MCT	11 (32)	4 (2)	13 (31)	**0.023**	13 (39)	7 (4)	14 (35)	0.069
L-carnitine	12 (36)	14 (8)	12 (28)	0.324	11 (32)	7 (4)	12 (28)	0.149
Sodium bicarbonate	15 (44)	32 (18)	11 (26)	**<0.001**	10 (30)	14 (8)	9 (22)	0.144
CLA	4 (13)	4 (2)	5 (11)	0.370	6 (19)	9 (5)	6 (14)	0.199
Herbs	4 (11)	0 (0)	5 (11)	**0.049**	5 (16)	2 (1)	6 (15)	0.086
Other	5 (14)	4 (2)	5 (12)	0.311	3 (10)	4 (2)	3 (8)	0.480

Significant differences are expressed in bold font.
